# Policy characteristics facilitating primary health care in Thailand: A pilot study in transitional country

**DOI:** 10.1186/1475-9276-8-8

**Published:** 2009-03-26

**Authors:** Krit Pongpirul, Barbara Starfield, Supattra Srivanichakorn, Supasit Pannarunothai

**Affiliations:** 1 Department of International Health, Johns Hopkins Bloomberg School of Public Health, Baltimore, MD, USA; 2 Department of Preventive and Social Medicine, Faculty of Medicine, Chulalongkorn University, Bangkok, Thailand; 3 Department of Health Policy and Management, Johns Hopkins Bloomberg School of Public Health, Baltimore, MD, USA; 4 Institute of Community based Health Care Research and Development, Ministry of Public Health, Nonthaburi, Thailand; 5 Department of Community, Family and Occupational Medicine, Faculty of Medicine, Naresuan University, Phitsanulok, Thailand

## Abstract

**Background:**

In contrast to the considerable evidence of inequitable distribution of *health*, little is known about how health *services (particularly primary care services) *are distributed in less developed countries. Using a version of primary health care system questionnaire, this pilot study in Thailand assessed policies related to the provision of primary care, particularly with regard to attempts to distribute resources equitably, adequacy of resources, comprehensiveness of services, and co-payment requirement. Information on other main attributes of primary health care policy was also ascertained.

**Methods:**

Questionnaire survey of 5 policymakers, 5 academicians, and 77 primary care practitioners who were attending a workshop on primary care. Descriptive statistics with Fischer's exact test were used for data analysis.

**Results:**

All policymakers and academicians completed the mailed questionnaire; the response rate among the practitioners was 53.25% (41 out of 77). However, the responses from all three groups were consistent in reporting that (1) financial resources were allocated based on different health needs and special efforts were made to assure primary care services to the needy or underserved population, (2) the supply of essential drugs was adequate, (3) clinical services were distributed equitably, (4) out-of-pocket payment was low, and that some primary health care attributes, particularly longitudinality (patients are seen by same doctor or team each time they make a visit), coordination, and family- and community-orientation were satisfactory. Geographical variations were present, suggesting inequitable distribution of primary care across regions. The questionnaire was robust across key stakeholders and feasible for use in a transitional country.

**Conclusion:**

A primary care systems questionnaire administered to different types of health professionals was able to show that resource distribution was equitable at a national level but some aspects of primary care practice across regions is still of concern, in at least in this transitional country.

## Background

Primary health care is a system-wide approach to designing health services based on primary care, which is regarded as a means to help reduce medical expenditures and provide more effective and equitable care to populations [1,2]. Equitable distribution of primary care services has been investigated in industrialized countries, mainly based on data from national surveys [1,3]. As developing countries are more likely to have inequitable access to health services, it is important to assess the extent to which new health policies improve the situation.

Starfield introduced an approach to compare primary health care policy and primary care practice characteristics, using document review and national expert interview to score primary health care of selected industrialized nations [4]. Based on pre-defined criteria, each of 13 policy and 7 practice characteristics were assigned a score from 0 (connoting the absence or poor development of the characteristics) to 2 (connoting a high level of development of the characteristics) [4]. Subsequent research [1] demonstrated that four of the systems policy characteristics (attempt to distribute resources equitably, adequacy of resources in primary care facilities, comprehensiveness of services, and low or no copayment) are most important in distinguishing health systems that have strong primary care orientation from those that do not.

The extent to which a similar approach would be useful in identifying differences in primary health care within countries with relatively greater resource limitations is unknown. For instance, relative shortages of physicians in different areas of these countries raises the question of whether having physicians at primary care facilities makes any difference. Moreover, prior studies have not determined the robustness of responses across various types of stakeholders: policymakers, academicians, and practitioners.

Thailand is a transitional country with approximately 65 million populations. In 2001, the Thai government introduced a policy of universal coverage (UC), to include 75% of total population [5] not covered by formal public insurance schemes. The policy is tax funded and has incorporated two main reform initiatives – reform of budget allocation and payment methods and strengthening primary care [6]. After the reform, the increased proportion of health service utilization at primary care level and community hospitals was claimed to reflect a success of the policy[5]. However, some problems such as physical access and travel costs still exist [7,8] whereas inequitable distribution of resources in general has still been a great concern [9].

This study in Thailand is aimed to assess important primary health care policy characteristics as well as the other attributes of primary health care in order to serve as a baseline for future policy changes.

## Methods

### Questionnaire

The original questions were developed for cross-national comparisons of primary care in OECD countries [10] and later modified to characterize aspects of national policy that influence the provision of primary care services. To better capture information in the Thai context, the questionnaire was modified and translated into Thai language and then backward translation was carried out to ensure accuracy. Face validity was assessed by 3 faculty members of the Faculty of Medicine of Chulalongkorn University who have expertise in primary care and community medicine. The questionnaire was piloted among 5 health policy graduate students for additional feedback regarding appropriateness of wording.

The modified questionnaire contains two major sections: characteristics of the respondents and 9 primary care attributes that include Resource Allocation, Adequacy of Resources, Copayment Requirements, Comprehensiveness of Care, First Contact, Longitudinality, Coordination, Family-Centeredness, Community Orientation, and Professional Personnel (Table 1).

**Table 1:** Primary Health Care Domains in the Questionnaire

RESOURCE ALLOCATION					
1. To what extent does the national government use differences in health needs as a basis for allocating money to different areas of the country?					
() Very little					
() To some extent					
() To a major extent, but not the most important determinant					
() The most important determinant					
Does the national government make special efforts to assure primary health care services to especially needy or underserved segments of the population (e.g., mobile health teams are organized to visit poor rural villages periodically)?					
() Yes, generally			() To some extent only		
() To a very small extent only			() No		
ADEQUACY OF RESOURCES					
1. In your estimation, for what percentage of the population covered by these primary health care facilities is there an adequate supply of essential drugs a majority of the time (e.g. common antibiotics, pain relievers, other medicines identified as needed)?					
() None	() 1–10%	() 11–20%	() 21–50%	() 51–75%	() 76–100%
2. In your estimation, for what percentage of the population covered by these primary health care facilities is there sufficient basic equipment and/or supplies to fulfill their functions adequately (e.g., a working sterilizer, needles for vaccinations, other basic equipment?)					
() None	() 1–10%	() 11–20%	() 21–50%	() 51–75%	() 76–100%

FIRST CONTACT					
1. Is consultation with a provider at the primary health care level required before someone is allowed to seek other care (e.g., in a hospital clinic, walk-in outpatient department, or specialist consultation) (except in cases of emergency)?					
() Definitely	() Mostly	() Mostly Not	() Definitely Not		

COPAYMENT REQUIREMENTS					
1. What percentage of primary health care facilities requires people to pay out-of-pocket at the point of services?					
() None	() 1–10%	() 11–20%	() 21–50%	() 51–75%	() 76–100%

LONGITUDINALITY					
1. Are patients generally seen by the same doctor or team each time they make a visit?					
() Definitely	() Mostly	() Mostly Not	() Definitely Not		
2. Is there a policy to enroll people within a geographic area with a specific primary health care provider or provider group, by keeping patient lists or rosters?					
() Definitely	() Mostly	() Mostly Not	() Definitely Not		
3. Is there a policy to ensure that primary health care facilities are regularly staffed by a physician or nurse?					
() Definitely	() Mostly	() Mostly Not	() Definitely Not		

COMPREHENSIVENESS OF CARE					
To what extent do primary health care facilities or practices deliver each of the following services?					
() Definitely	() Mostly	() Mostly Not	() Definitely Not		
1. Vaccinations for children					
2. Illnesses care for children					
3. Illnesses care for adults					
4. Illnesses care for the elderly					
5. Prenatal care/safe delivery					
6. Family planning services					
7. Care of sexually transmitted diseases					
8. Treatment of tuberculosis					
9. Treatment of minor injuries					
10. Counseling about alcohol and tobacco use					
11. Minor surgery					
12. Non-major mental health problems					
13. Care for chronic illness					
14. Health education					
15. Screening/treatment of parasitic disease(s)					
16. Nutrition program					
17. School-based services					

COORDINATION					
1. In the primary health care system, is there a requirement to use a growth monitoring and vaccination record for all children seen in primary health care facilities?					
() Definitely	() Mostly	() Mostly Not	() Definitely Not		
2. Is there a client-held record of vaccinations and growth monitoring?					
() Definitely	() Mostly	() Mostly Not	() Definitely Not		
3. Is there a requirement to use a Prenatal Control Record?					
() Definitely	() Mostly	() Mostly Not	() Definitely Not		
4. Is there a client-held record of prenatal care visits and test results for all women seen during pregnancy?					
() Definitely	() Mostly	() Mostly Not	() Definitely Not		
5. Are there formal guidelines or common practice for transfer of information between the primary health care level and other levels of the health care system?					
() Definitely	() Mostly	() Mostly Not	() Definitely Not		

FAMILY-CENTEREDNESS					
1. Are charts at primary health care facilities arranged by family (rather than by individual)?					
() Definitely	() Mostly	() Mostly Not	() Definitely Not		

COMMUNITY ORIENTATION					
Does the health facility:					
1. Conduct surveys of patients to see if the services are meeting people's needs?					
() Definitely	() Mostly	() Mostly Not	() Definitely Not		
2. Conduct surveys in the community to find about health problems they should know about?					
() Definitely	() Mostly	() Mostly Not	() Definitely Not		
3. Ask family members to be on the Board of Directors or advisory committee?					
() Definitely	() Mostly	() Mostly Not	() Definitely Not		

PROFESSIONAL PERSONNEL					
1. Do nurses serve as primary health care practitioners, that is, substitute for physicians?					
() Definitely	() Mostly	() Mostly Not	() Definitely Not		
2. Indicate what type of health worker most commonly staffs most primary health care facilities (excluding hospital clinics)					
○ Community health workers only					
○ Community health workers and nurse(s)					
○ Community health workers, nurse(s), and physician(s)					
○ Nurse(s) only					
○ Physician(s) and nurse(s)					
○ Physician(s) only					
3. Do medical students at medical schools receive training in primary health care?					
() Definitely	() Mostly	() Mostly Not	() Definitely Not		

### Respondents

While 'national experts' in the original approach were selectively identified by the investigator based on personal knowledge of individuals in the countries [4], this study sought key players in the Thai health care system at national and provincial levels. Primary Health Care Policymakers are those who have policy-making, supervisory, or regulatory roles for primary care. They were identified by snowballing technique, starting from one of the investigator (SS). Primary Care Academicians are key contributors to various aspects of primary care knowledge in Thailand, identified from having primary care-focused publications in Thai and/or international journals. Primary Care Practitioners were providers from 13 provinces in 4 regions who attended the government-funded workshop on primary care development on July 17–18, 2007. The questionnaire was distributed in the first day and the respondents were asked to complete and return them the next day.

### Data Analysis

Descriptive statistics were used to analyze policy characteristics and differences across geographical areas, provinces, and staffing pattern (have doctor vs have no doctor). Given small sample size, Fisher's exact test was used to analyze the differences across respondent groups and regions. Staffing pattern of primary care facilities as with and without on-site physicians was analyzed for each domain using Fisher's exact test.

## Results

### Characteristics of Respondents

Five policymakers (mean age 52 years; all male), five academicians (mean age 45 years; one male) and forty-one practitioners (mean age 44 years; 8 male) were recruited. Response rates for the first two groups were 100% whereas 53.25% of the practitioners returned the questionnaire (Table 2). The relatively low response rate was because practitioners from some provinces attended only the first day of the workshop and did not return the questionnaire on the second day as requested. The respondents had been providing primary care for approximately 15 years on average (range 1–37 years). The proportion of physician, nurse, and other health care professionals in this convenience sample is comparable to that of the country. As there were few significant differences among respondent groups, the findings are presented by combining all three types of respondents where appropriate.

**Table 2:** Responses from primary care practitioners

**Region**	**Province**	Attend*	**Response**
North	Phrae	7	0

(5/17)	Phitsanulok	4	0

	Nakhon Sawan	6	5

Central	Ayudhaya	6	1

(10/19)	Ratchaburi	4	0

	Chachoengsao	9	9

Northeast	Roi-Et	8	5

(14/20)	Kalasin	5	2

	Nakhonratchasima	4	4

	Srisaket	3	3

South	Chumporn	4	0

(12/21)	Nakhonsrithammarat	9	6

	Songkhla	8	6

	Total	77	41

* Based on the list of attendees for the primary care development workshop on July 17–18, 2007.

### Equitable distribution of resources

More than four out of five responses reported that the government makes a major effort to distribute resources equitably; only one in twenty reported that little attempt is made to do so.

Almost three quarters of respondents reported that the government makes special efforts to assure primary care services to especially needy or underserved segments of the population. While there was no significant difference among respondent groups (Fisher's exact p = 0.273), there was some evidence of regional variation (Fisher's exact p = 0.053).

### Adequacy of facilities

A majority of respondents (56%) agreed that at least three-quarters of the population have an adequate supply of essential drugs. The response was uniform across regions (Fisher's exact p = 0.15) but one respondent from Northeastern region and two respondents from Southern region reported that less than one in five has access to an adequate supply of essential drugs.

About four in five of the respondents reported that more than three-quarters of the health facilities have sufficient basic equipment and/or supplies to fulfil their functions adequately. Regional variation is very significant (Fisher's exact p = 0.002). Half of the respondents from the Southern region reported that less than half of the population receive care from primary care facilities with sufficient basic equipment and supply.

### Comprehensiveness

**Figure 1:**
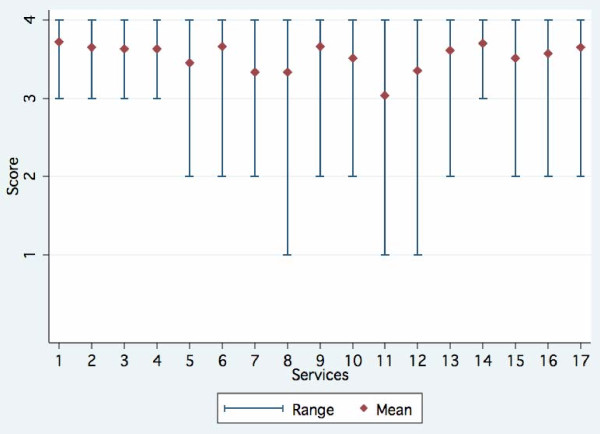
**Comprehensiveness of Primary Care Services**. NB. The horizontal access refers to the different services as indicated in the questionnaire items. This graph shows the 4-scale responses (1, Definitely Not; 2, Mostly Not; 3, Mostly; 4, Definitely) to the question "To what extent do primary health care facilities or practices deliver each of the following services?" : (1) Vaccinations for children, (2) Illnesses care for children, (3) Illnesses care for adults, (4) Illnesses care for the elderly, (5) Prenatal care/safe delivery, (6) Family planning services, (7) Care of sexually transmitted diseases, (8) Treatment of tuberculosis, (9) Treatment of minor injuries, (10) Counseling about alcohol and tobacco use, (11) Minor surgery, (12) Non-major mental health problems, (13) Care for chronic illness, (14) Health education, (15) Screening/treatment of parasitic diseases, (16) Nutrition program, (17) School-based services.

Comprehensiveness refers to "the extent to which primary care practitioners provided a broad range of services rather than making referrals to specialists for those services" [2]. In general, the respondents agree that most preventive services are adequately delivered. The coverage of some curative services that can reduce excessive use of specialized care for children and elderly seems satisfactory. However, some facilities are unable to offer some curative care such as minor surgery (#11), care for non-major mental health problems (#12), and treatment of tuberculosis (#8) (Figure 1).

### Co-payment Requirement

Almost three of four respondents concur that few (less than one in ten primary care facilities) require people to pay out-of-pocket at the point of services whereas only one in five respondents report that up to one-fifth of primary care facilities require out-of-pocket payment. The responses are not different across groups (Fisher's exact p = 0.114) but two of the five policymakers reported that out-of-pocket payment is required in as high as a half of primary care facilities.

### Other main attributes of primary care

#### First Contact

A large majority of respondents (78.43%) reported that consultation with a provider at the primary care level is required before someone is allowed to seek other care (e.g., in a hospital clinic, walk-in outpatient department, or specialist consultation), except in cases of emergency. However, one in four practitioners from regions other than the North do not agree. Likewise, 4 out of 10 practitioners from the Central region reported that such a consultation is not required. This inconsistent response within the province warrants further exploration of the issue.

#### Longitudinality

Generally, patients are mostly seen by same doctor or team each time they make a visit. However, variation across regions may exist (Fisher's exact p = 0.062), especially in the Southern region, in which 42% do not agree.

There is a policy to enrol people within a geographic area with a specific primary care provider, especially in the Northeastern region. There is no significant variation by region (Fisher's exact p = 0.263). Similarly, the respondents agree that there is a policy to ensure that primary care facilities are regularly staffed by a physician or nurse (Fisher's exact p = 0.967).

#### Coordination

All respondents agree that essential tools for coordination are in place in the primary health care system. These include procedures for growth monitoring, vaccination, and prenatal record. Formal guidelines for transfer of information from primary care to other levels do exist.

#### Family-Centeredness

Family-centeredness is "the degree to which services were provided to all family members by the same practitioner" [2]. All respondents reported that people's health records at primary care facilities are arranged by family rather than by individual.

#### Community Orientation

A majority of respondents concur that health facilities in each area conduct surveys of patients and community to see if the services are meeting people's needs and that family members are asked to become committee members at the primary care facility. However, there is variation by type of respondent (Fisher's exact p = 0.019) as 60% of the academicians do not think that patient surveys are really in place.

#### Professional Personnel

Using nurses as primary care practitioners is reported as common across regions (Fisher's exact p = 0.142). A majority of respondents (96%) reported that medical students receive training in primary care and primary health care at medical schools.

The most common staffing pattern at primary care facilities is having health workers, nurses, and doctors (48%) but 46% of the facilities have no doctor. The presence of at least one physician at a primary care facility is not associated with the score on any of the domains, and only one item (screening/treatment of parasitic disease) shows a significant p value.

#### Regional variation

There was little regional variation, although two domains (First Contact and Longitudinalty) were worse in the Central and the Southern regions, suggesting a need for attention there. It was also the case that the Southern region lacks some essential services, such as treatment of tuberculosis and minor surgery.

## Discussion

This study was the first to pilot a simple approach to assess primary health care in a country with a limited database and national expertise in primary care evaluation. Three major stakeholders were surveyed and some opinion gaps were expected. However, the response was found to be robust with some minor exceptions. Some primary care domains at a clinical level, particularly longitudinality, coordination, and family- and community-orientation were found to be satisfactory whereas the others may need more attention.

The PCAT has been used in many countries at the individual patient or facilities level rather than at the system level that was the focus in this pilot study. Cassady et al (2000) found the PCAT to be valid and reliable (Cronbach's α ranging from 0.68 to 0.86 for the domains) [11]. In Brazil, Harzheim et al (2006) reported similar findings with a translated PCAT (Cronbach's α 0.74 – 0.88) [12]. Recent validation studies in Spain and Canada found similar results [13,14]. Although similar psychometric property can be expected from this modified PCAT for system assessment, further validation and reliability study is warranted.

Since 2001, the Universal Health Care policy has been implemented in Thailand, aiming to increase equity in health care service among the Thai population regardless of their financial situation [15]. Despite improved access, top-level executives of the Ministry of Public Health still expressed concern about inequitable distribution of resources in general [9]. Our study examined the issue and found that geographical inequity does exist, especially for the supply of equipment in the Southern and Northeastern region. Although this discrepancy could be solved by better budget allocation, the current capitation calculation that is based on national average does not accommodate this concern. Further study is required to explore this issue.

Financial barriers to primary care access seem to be only partially solved by the introduction of the reform as suggested by our findings that copayment still exists and should be further investigated. Unfortunately, conducting such a study might be too late as the current budget shortfall has driven the possibility of increased copayment requirement in the near future. A new study on exploring various alternatives for cost sharing is therefore being proposed [16]. The system should be reassessed once copayment is introduced.

It has been previously claimed that the recent health system reform provides comprehensive health care benefit [15]. Our study is one of the very few that attempts to explore this domain by using 17 key primary care services essential to a transitional country like Thailand. Our finding, however, suggests that primary care services in Thailand are not equitably comprehensive, as some important services are not adequately provided in some areas. Rojpibulstit et al (2006) reported that some logistics introduced by the reform might indeed cause the delay in seeking good primary care [17]. They suggested that the referral requirement from lower- to higher-level health facilities might be a reason for delays in the treatment of tuberculosis in the Southern region, which has much poorer supply of health professionals and medical facilities.

Issues concerning the clinical quality of primary care are more difficult. A study found that basic psychiatric services are provided by inexperienced general practitioners in primary care settings with inadequate supply of new and appropriate drugs [18]. Another study reported that routine diabetic assessments were not regularly done in primary care units [19]. This situation poses an important challenge for all health systems: which services should be considered as of high enough frequency to be provided in primary care in various regions, given resource constraints?

While an increasing literature from OECD countries focuses on the relationship between the supply and deployment of primary care physicians and health outcomes, application of those published findings might be less relevant in less developed countries [20]. In Thailand, nurses and paramedics play a major role in primary care as they are present in 98% of the primary care facilities whereas only 46% have physician availability. Our finding suggested that the continuous presence of physicians might not make any difference to service provision; however, further study with larger sample size and more focus on health outcomes should be conducted.

Our study has some limitations. First, ascertaining experiences of the population with its health system is crucial [4]. Although it was beyond the scope of this pilot study, instruments are available to accomplish this [21]. Second, convenience sampling techniques used for recruitment of respondents might limit generalizability of this finding. However, we believe it is an appropriate approach for less developed countries with limited resources. Third, the small sample size and low response rate may prevent policy-related application.

## Conclusion

This pilot study indicated that the questionnaire was robust across key stakeholders and feasible for a transitional country. Resource distribution was found to be equitable at national level but primary care clinical practice across regions is still of concern. The next step is to apply and validate the tool to a larger sample in various settings in Thailand as well as other developing countries.

## Competing interests

The authors declare that they have no competing interests.

## Authors' contributions

KP conceived of the study, participated in its design, carried out the survey, analyzed the data, and drafted the manuscript. BS conceived of the study, participated in its design, and helped to draft the manuscript. SS participated in its design, helped to carry out the survey. SP helped to draft the manuscript. All authors read and approved the final manuscript.
